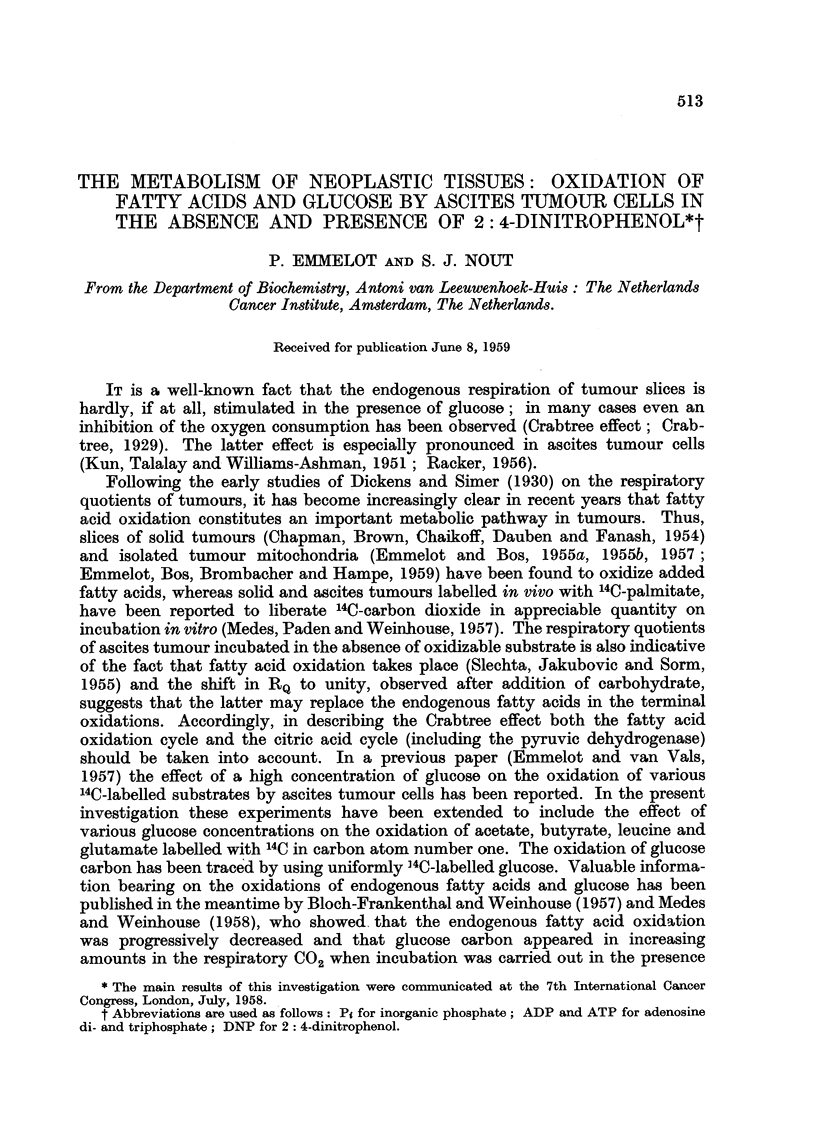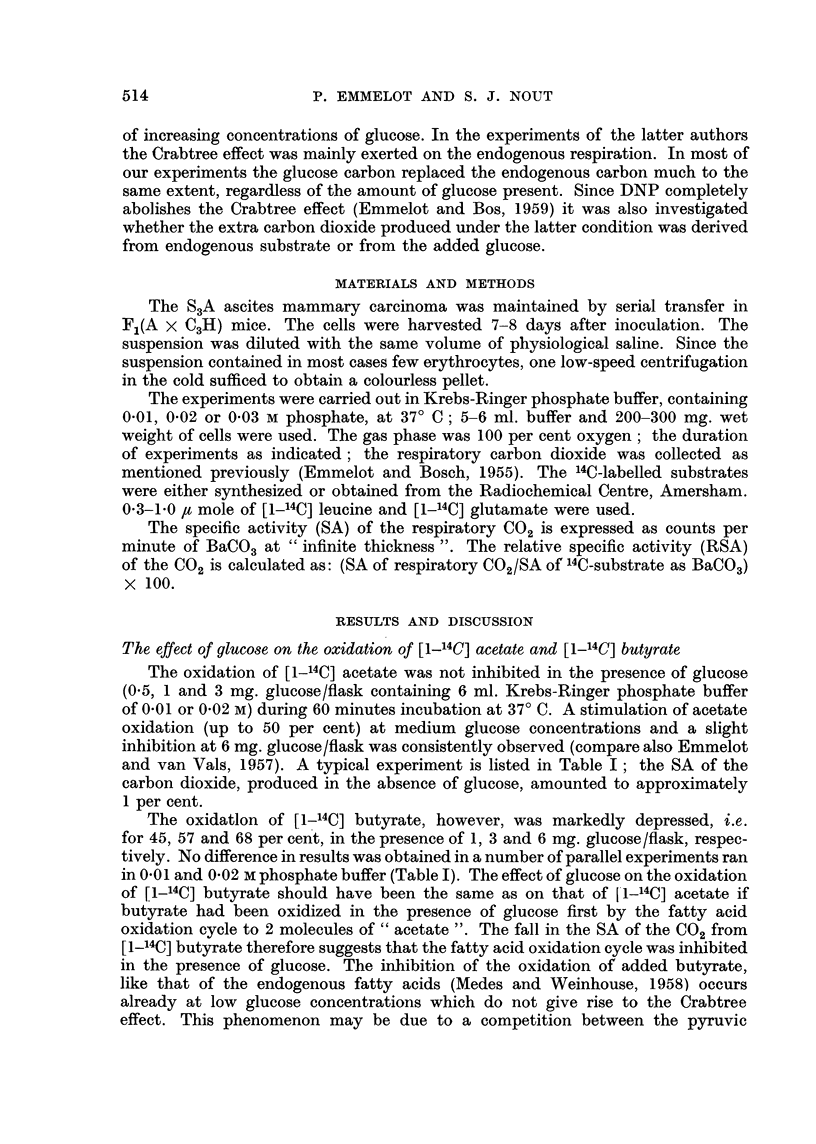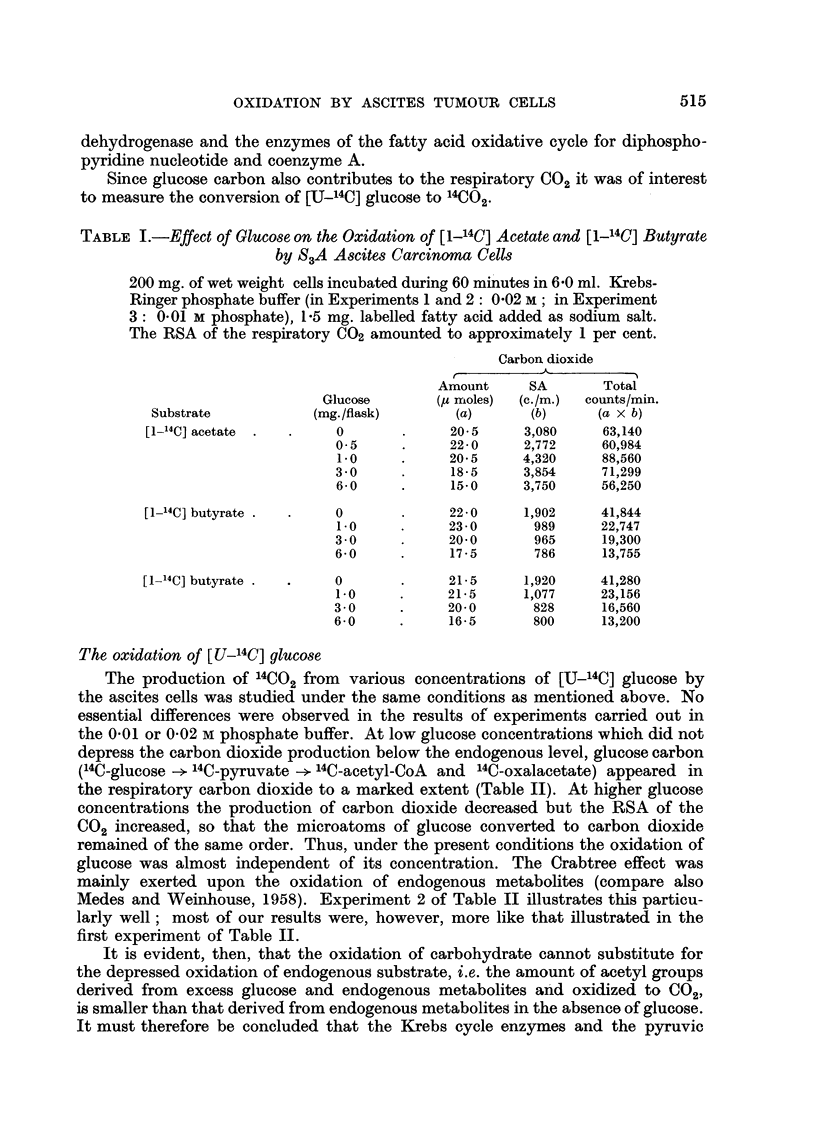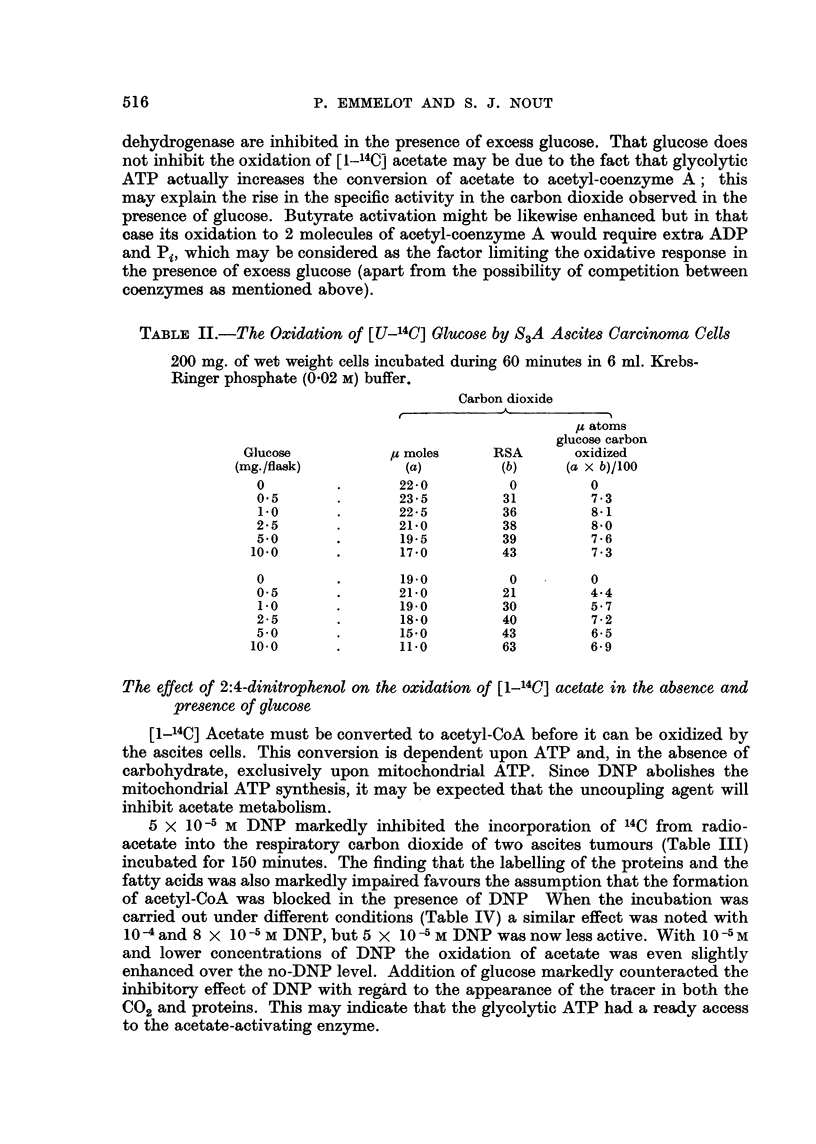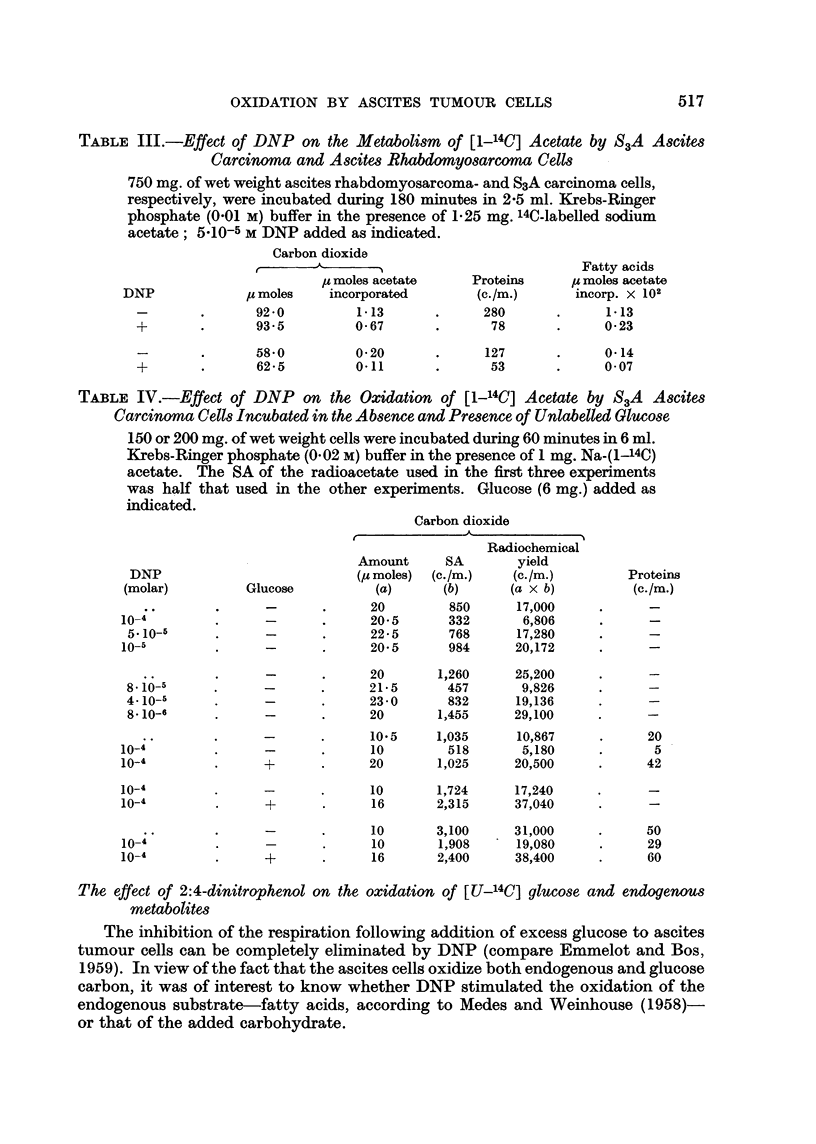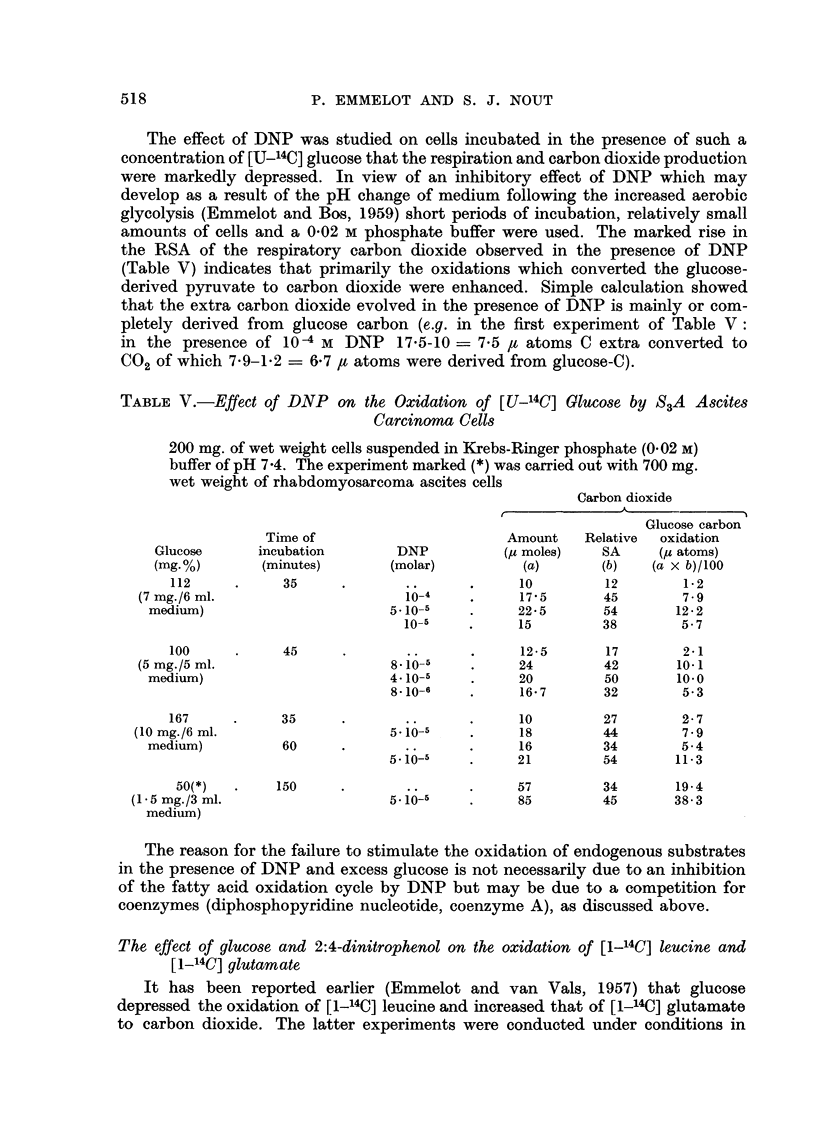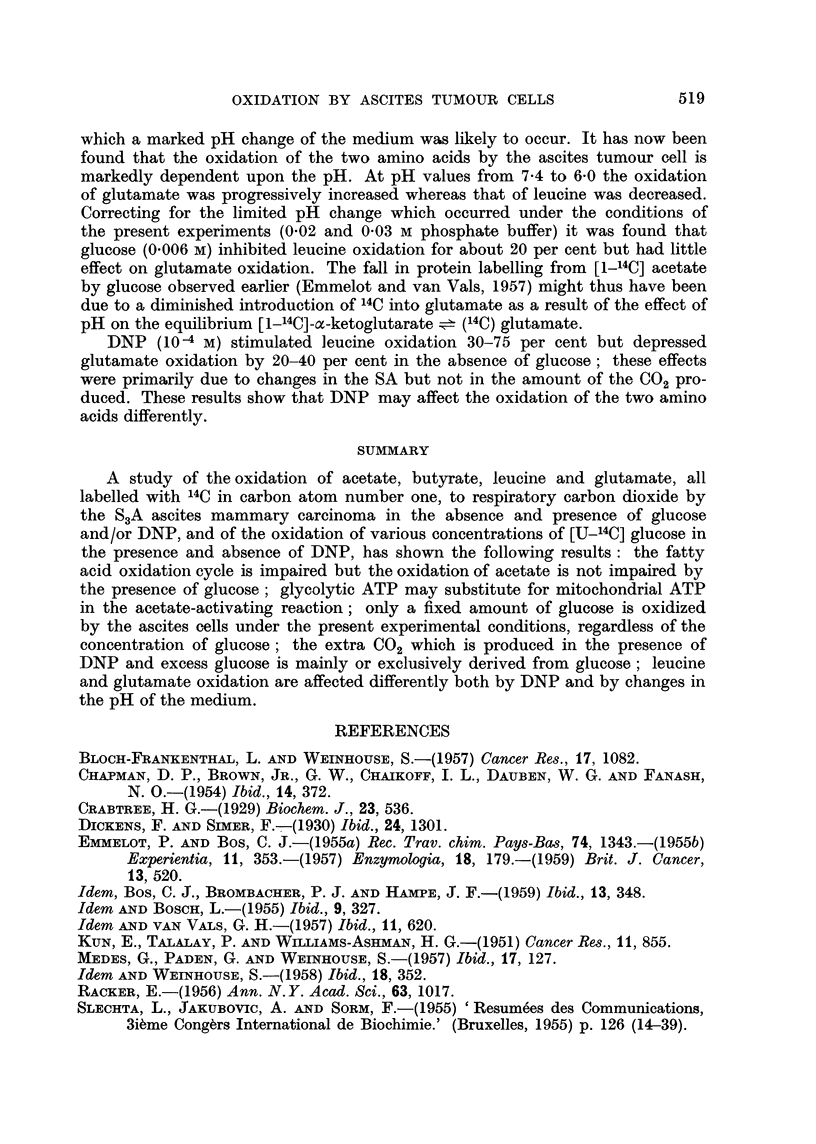# The Metabolism of Neoplastic Tissues: Oxidation of Fatty Acids and Glucose by Ascites Tumour Cells in the Absence and Presence of 2:4-Dinitrophenol*,†

**DOI:** 10.1038/bjc.1959.57

**Published:** 1959-09

**Authors:** P. Emmelot, S. J. Nout


### THE METABOLISM OF NEOPLASTIC TISSUES: OXIDATION OF FATTY ACIDS AND GLUCOSE BY ASCITES TUMOUR CELLS IN THE ABSENCE AND PRESENCE OF 2:4-DINITROPHENOL*†

P. EMMELOT AND S. J. NOUT

*From the Department of Biochemistry, Antoni van Leeuwenhoek-Huis : The Netherlands Cancer Institute, Amsterdam, The Netherlands.*

Received for publication June 8, 1959

IT is a well-known fact that the endogenous respiration of tumour slices is hardly, if at all, stimulated in the presence of glucose ; in many cases even an inhibition of the oxygen consumption has been observed (Crabtree effect ; Crabtree, 1929). The latter effect is especially pronounced in ascites tumour cells (Kun, Talalay and Williams-Ashman, 1951 ; Racker, 1956).

Following the early studies of Dickens and Simer (1930) on the respiratory quotients of tumours, it has become increasingly clear in recent years that fatty acid oxidation constitutes an important metabolic pathway in tumours. Thus, slices of solid tumours (Chapman, Brown, Chaikoff, Dauben and Fanash, 1954) and isolated tumour mitochondria (Emmelot and Bos, 1955*a*, 1955*b*, 1957 ; Emmelot, Bos, Brombacher and Hampe, 1959) have been found to oxidize added fatty acids, whereas solid and ascites tumours labelled *in vivo* with $^{14}C$-palmitate, have been reported to liberate $^{14}C$-carbon dioxide in appreciable quantity on incubation *in vitro* (Medes, Paden and Weinhouse, 1957). The respiratory quotients of ascites tumour incubated in the absence of oxidizable substrate is also indicative of the fact that fatty acid oxidation takes place (Slechta, Jakubovic and Sorm, 1955) and the shift in $R_Q$ to unity, observed after addition of carbohydrate, suggests that the latter may replace the endogenous fatty acids in the terminal oxidations. Accordingly, in describing the Crabtree effect both the fatty acid oxidation cycle and the citric acid cycle (including the pyruvic dehydrogenase) should be taken into account. In a previous paper (Emmelot and van Vals, 1957) the effect of a high concentration of glucose on the oxidation of various $^{14}C$-labelled substrates by ascites tumour cells has been reported. In the present investigation these experiments have been extended to include the effect of various glucose concentrations on the oxidation of acetate, butyrate, leucine and glutamate labelled with $^{14}C$ in carbon atom number one. The oxidation of glucose carbon has been traced by using uniformly $^{14}C$-labelled glucose. Valuable information bearing on the oxidations of endogenous fatty acids and glucose has been published in the meantime by Bloch-Frankenthal and Weinhouse (1957) and Medes and Weinhouse (1958), who showed that the endogenous fatty acid oxidation was progressively decreased and that glucose carbon appeared in increasing amounts in the respiratory $CO_2$ when incubation was carried out in the presence

* The main results of this investigation were communicated at the 7th International Cancer Congress, London, July, 1958.

† Abbreviations are used as follows : $P_i$ for inorganic phosphate ; ADP and ATP for adenosine di- and triphosphate ; DNP for 2 : 4-dinitrophenol.

of increasing concentrations of glucose. In the experiments of the latter authors the Crabtree effect was mainly exerted on the endogenous respiration. In most of our experiments the glucose carbon replaced the endogenous carbon much to the same extent, regardless of the amount of glucose present. Since DNP completely abolishes the Crabtree effect (Emmelot and Bos, 1959) it was also investigated whether the extra carbon dioxide produced under the latter condition was derived from endogenous substrate or from the added glucose.

### MATERIALS AND METHODS

The $S_3A$ ascites mammary carcinoma was maintained by serial transfer in $F_1(A \times C_3H)$ mice. The cells were harvested 7–8 days after inoculation. The suspension was diluted with the same volume of physiological saline. Since the suspension contained in most cases few erythrocytes, one low-speed centrifugation in the cold sufficed to obtain a colourless pellet.

The experiments were carried out in Krebs-Ringer phosphate buffer, containing 0·01, 0·02 or 0·03 M phosphate, at 37° C ; 5–6 ml. buffer and 200–300 mg. wet weight of cells were used. The gas phase was 100 per cent oxygen ; the duration of experiments as indicated ; the respiratory carbon dioxide was collected as mentioned previously (Emmelot and Bosch, 1955). The $^{14}C$-labelled substrates were either synthesized or obtained from the Radiochemical Centre, Amersham. 0·3–1·0 $\mu$ mole of [1–$^{14}C$] leucine and [1–$^{14}C$] glutamate were used.

The specific activity (SA) of the respiratory $CO_2$ is expressed as counts per minute of $BaCO_3$ at " infinite thickness ". The relative specific activity (RSA) of the $CO_2$ is calculated as: (SA of respiratory $CO_2$/SA of $^{14}C$-substrate as $BaCO_3$) $\times$ 100.

### RESULTS AND DISCUSSION

*The effect of glucose on the oxidation of* [1–$^{14}C$] *acetate and* [1–$^{14}C$] *butyrate*

The oxidation of [1–$^{14}C$] acetate was not inhibited in the presence of glucose (0·5, 1 and 3 mg. glucose/flask containing 6 ml. Krebs-Ringer phosphate buffer of 0·01 or 0·02 M) during 60 minutes incubation at 37° C. A stimulation of acetate oxidation (up to 50 per cent) at medium glucose concentrations and a slight inhibition at 6 mg. glucose/flask was consistently observed (compare also Emmelot and van Vals, 1957). A typical experiment is listed in Table I ; the SA of the carbon dioxide, produced in the absence of glucose, amounted to approximately 1 per cent.

The oxidatlon of [1–$^{14}C$] butyrate, however, was markedly depressed, *i.e.* for 45, 57 and 68 per cent, in the presence of 1, 3 and 6 mg. glucose/flask, respectively. No difference in results was obtained in a number of parallel experiments ran in 0·01 and 0·02 M phosphate buffer (Table I). The effect of glucose on the oxidation of [1–$^{14}C$] butyrate should have been the same as on that of [1–$^{14}C$] acetate if butyrate had been oxidized in the presence of glucose first by the fatty acid oxidation cycle to 2 molecules of " acetate ". The fall in the SA of the $CO_2$ from [1–$^{14}C$] butyrate therefore suggests that the fatty acid oxidation cycle was inhibited in the presence of glucose. The inhibition of the oxidation of added butyrate, like that of the endogenous fatty acids (Medes and Weinhouse, 1958) occurs already at low glucose concentrations which do not give rise to the Crabtree effect. This phenomenon may be due to a competition between the pyruvic dehydrogenase and the enzymes of the fatty acid oxidative cycle for diphosphopyridine nucleotide and coenzyme A.

Since glucose carbon also contributes to the respiratory $CO_2$ it was of interest to measure the conversion of [U–$^{14}$C] glucose to $^{14}CO_2$.

TABLE I.—*Effect of Glucose on the Oxidation of [1–$^{14}$C] Acetate and [1–$^{14}$C] Butyrate by $S_3A$ Ascites Carcinoma Cells*

200 mg. of wet weight cells incubated during 60 minutes in 6·0 ml. Krebs-Ringer phosphate buffer (in Experiments 1 and 2 : 0·02 M ; in Experiment 3 : 0·01 M phosphate), 1·5 mg. labelled fatty acid added as sodium salt. The RSA of the respiratory $CO_2$ amounted to approximately 1 per cent.

| Substrate | Glucose (mg./flask) | Carbon dioxide: Amount (μ moles) (a) | Carbon dioxide: SA (c./m.) (b) | Carbon dioxide: Total counts/min. (a × b) |
|---|---|---|---|---|
| [1–$^{14}$C] acetate | 0 | 20·5 | 3,080 | 63,140 |
| | 0·5 | 22·0 | 2,772 | 60,984 |
| | 1·0 | 20·5 | 4,320 | 88,560 |
| | 3·0 | 18·5 | 3,854 | 71,299 |
| | 6·0 | 15·0 | 3,750 | 56,250 |
| [1–$^{14}$C] butyrate | 0 | 22·0 | 1,902 | 41,844 |
| | 1·0 | 23·0 | 989 | 22,747 |
| | 3·0 | 20·0 | 965 | 19,300 |
| | 6·0 | 17·5 | 786 | 13,755 |
| [1–$^{14}$C] butyrate | 0 | 21·5 | 1,920 | 41,280 |
| | 1·0 | 21·5 | 1,077 | 23,156 |
| | 3·0 | 20·0 | 828 | 16,560 |
| | 6·0 | 16·5 | 800 | 13,200 |

*The oxidation of [U–$^{14}$C] glucose*

The production of $^{14}CO_2$ from various concentrations of [U–$^{14}$C] glucose by the ascites cells was studied under the same conditions as mentioned above. No essential differences were observed in the results of experiments carried out in the 0·01 or 0·02 M phosphate buffer. At low glucose concentrations which did not depress the carbon dioxide production below the endogenous level, glucose carbon ($^{14}$C-glucose → $^{14}$C-pyruvate → $^{14}$C-acetyl-CoA and $^{14}$C-oxalacetate) appeared in the respiratory carbon dioxide to a marked extent (Table II). At higher glucose concentrations the production of carbon dioxide decreased but the RSA of the $CO_2$ increased, so that the microatoms of glucose converted to carbon dioxide remained of the same order. Thus, under the present conditions the oxidation of glucose was almost independent of its concentration. The Crabtree effect was mainly exerted upon the oxidation of endogenous metabolites (compare also Medes and Weinhouse, 1958). Experiment 2 of Table II illustrates this particularly well ; most of our results were, however, more like that illustrated in the first experiment of Table II.

It is evident, then, that the oxidation of carbohydrate cannot substitute for the depressed oxidation of endogenous substrate, *i.e.* the amount of acetyl groups derived from excess glucose and endogenous metabolites and oxidized to $CO_2$, is smaller than that derived from endogenous metabolites in the absence of glucose. It must therefore be concluded that the Krebs cycle enzymes and the pyruvic dehydrogenase are inhibited in the presence of excess glucose. That glucose does not inhibit the oxidation of [1–$^{14}$C] acetate may be due to the fact that glycolytic ATP actually increases the conversion of acetate to acetyl-coenzyme A; this may explain the rise in the specific activity in the carbon dioxide observed in the presence of glucose. Butyrate activation might be likewise enhanced but in that case its oxidation to 2 molecules of acetyl-coenzyme A would require extra ADP and $P_i$, which may be considered as the factor limiting the oxidative response in the presence of excess glucose (apart from the possibility of competition between coenzymes as mentioned above).

TABLE II.—*The Oxidation of [U–$^{14}$C] Glucose by $S_3A$ Ascites Carcinoma Cells*

200 mg. of wet weight cells incubated during 60 minutes in 6 ml. Krebs-Ringer phosphate (0·02 M) buffer.

| Glucose (mg./flask) | Carbon dioxide: μ moles (a) | RSA (b) | μ atoms glucose carbon oxidized (a × b)/100 |
|---|---|---|---|
| 0 | 22·0 | 0 | 0 |
| 0·5 | 23·5 | 31 | 7·3 |
| 1·0 | 22·5 | 36 | 8·1 |
| 2·5 | 21·0 | 38 | 8·0 |
| 5·0 | 19·5 | 39 | 7·6 |
| 10·0 | 17·0 | 43 | 7·3 |
| | | | |
| 0 | 19·0 | 0 | 0 |
| 0·5 | 21·0 | 21 | 4·4 |
| 1·0 | 19·0 | 30 | 5·7 |
| 2·5 | 18·0 | 40 | 7·2 |
| 5·0 | 15·0 | 43 | 6·5 |
| 10·0 | 11·0 | 63 | 6·9 |

*The effect of 2:4-dinitrophenol on the oxidation of [1–$^{14}$C] acetate in the absence and presence of glucose*

[1–$^{14}$C] Acetate must be converted to acetyl-CoA before it can be oxidized by the ascites cells. This conversion is dependent upon ATP and, in the absence of carbohydrate, exclusively upon mitochondrial ATP. Since DNP abolishes the mitochondrial ATP synthesis, it may be expected that the uncoupling agent will inhibit acetate metabolism.

$5 \times 10^{-5}$ M DNP markedly inhibited the incorporation of $^{14}$C from radio-acetate into the respiratory carbon dioxide of two ascites tumours (Table III) incubated for 150 minutes. The finding that the labelling of the proteins and the fatty acids was also markedly impaired favours the assumption that the formation of acetyl-CoA was blocked in the presence of DNP When the incubation was carried out under different conditions (Table IV) a similar effect was noted with $10^{-4}$ and $8 \times 10^{-5}$ M DNP, but $5 \times 10^{-5}$ M DNP was now less active. With $10^{-5}$ M and lower concentrations of DNP the oxidation of acetate was even slightly enhanced over the no-DNP level. Addition of glucose markedly counteracted the inhibitory effect of DNP with regard to the appearance of the tracer in both the $CO_2$ and proteins. This may indicate that the glycolytic ATP had a ready access to the acetate-activating enzyme.

TABLE III.—*Effect of DNP on the Metabolism of* [1–$^{14}$C] *Acetate by* $S_3A$ *Ascites Carcinoma and Ascites Rhabdomyosarcoma Cells*

750 mg. of wet weight ascites rhabdomyosarcoma- and $S_3A$ carcinoma cells, respectively, were incubated during 180 minutes in 2·5 ml. Krebs-Ringer phosphate (0·01 M) buffer in the presence of 1·25 mg. $^{14}$C-labelled sodium acetate ; 5·10$^{-5}$ M DNP added as indicated.

| DNP | Carbon dioxide: μ moles | Carbon dioxide: μ moles acetate incorporated | Proteins (c./m.) | Fatty acids μ moles acetate incorp. × 10$^2$ |
|---|---|---|---|---|
| – | 92·0 | 1·13 | 280 | 1·13 |
| + | 93·5 | 0·67 | 78 | 0·23 |
| – | 58·0 | 0·20 | 127 | 0·14 |
| + | 62·5 | 0·11 | 53 | 0·07 |

TABLE IV.—*Effect of DNP on the Oxidation of* [1–$^{14}$C] *Acetate by* $S_3A$ *Ascites Carcinoma Cells Incubated in the Absence and Presence of Unlabelled Glucose*

150 or 200 mg. of wet weight cells were incubated during 60 minutes in 6 ml. Krebs-Ringer phosphate (0·02 M) buffer in the presence of 1 mg. Na-(1–$^{14}$C) acetate. The SA of the radioacetate used in the first three experiments was half that used in the other experiments. Glucose (6 mg.) added as indicated.

| DNP (molar) | Glucose | Carbon dioxide: Amount (μ moles) (a) | Carbon dioxide: SA (c./m.) (b) | Carbon dioxide: Radiochemical yield (c./m.) (a × b) | Proteins (c./m.) |
|---|---|---|---|---|---|
| .. | – | 20 | 850 | 17,000 | – |
| 10$^{-4}$ | – | 20·5 | 332 | 6,806 | – |
| 5·10$^{-5}$ | – | 22·5 | 768 | 17,280 | – |
| 10$^{-5}$ | – | 20·5 | 984 | 20,172 | – |
| .. | – | 20 | 1,260 | 25,200 | – |
| 8·10$^{-5}$ | – | 21·5 | 457 | 9,826 | – |
| 4·10$^{-5}$ | – | 23·0 | 832 | 19,136 | – |
| 8·10$^{-6}$ | – | 20 | 1,455 | 29,100 | – |
| .. | – | 10·5 | 1,035 | 10,867 | 20 |
| 10$^{-4}$ | – | 10 | 518 | 5,180 | 5 |
| 10$^{-4}$ | + | 20 | 1,025 | 20,500 | 42 |
| 10$^{-4}$ | – | 10 | 1,724 | 17,240 | – |
| 10$^{-4}$ | + | 16 | 2,315 | 37,040 | – |
| .. | – | 10 | 3,100 | 31,000 | 50 |
| 10$^{-4}$ | – | 10 | 1,908 | 19,080 | 29 |
| 10$^{-4}$ | + | 16 | 2,400 | 38,400 | 60 |

*The effect of* 2:4-*dinitrophenol on the oxidation of* [*U*–$^{14}$*C*] *glucose and endogenous metabolites*

The inhibition of the respiration following addition of excess glucose to ascites tumour cells can be completely eliminated by DNP (compare Emmelot and Bos, 1959). In view of the fact that the ascites cells oxidize both endogenous and glucose carbon, it was of interest to know whether DNP stimulated the oxidation of the endogenous substrate—fatty acids, according to Medes and Weinhouse (1958)—or that of the added carbohydrate.

The effect of DNP was studied on cells incubated in the presence of such a concentration of [U–$^{14}$C] glucose that the respiration and carbon dioxide production were markedly depressed. In view of an inhibitory effect of DNP which may develop as a result of the pH change of medium following the increased aerobic glycolysis (Emmelot and Bos, 1959) short periods of incubation, relatively small amounts of cells and a 0·02 M phosphate buffer were used. The marked rise in the RSA of the respiratory carbon dioxide observed in the presence of DNP (Table V) indicates that primarily the oxidations which converted the glucose-derived pyruvate to carbon dioxide were enhanced. Simple calculation showed that the extra carbon dioxide evolved in the presence of DNP is mainly or completely derived from glucose carbon (*e.g.* in the first experiment of Table V: in the presence of $10^{-4}$ M DNP 17·5-10 = 7·5 $\mu$ atoms C extra converted to $CO_2$ of which 7·9–1·2 = 6·7 $\mu$ atoms were derived from glucose-C).

TABLE V.—*Effect of DNP on the Oxidation of* [*U*–$^{14}$*C*] *Glucose by* $S_3A$ *Ascites Carcinoma Cells*

200 mg. of wet weight cells suspended in Krebs-Ringer phosphate (0·02 M) buffer of pH 7·4. The experiment marked (*) was carried out with 700 mg. wet weight of rhabdomyosarcoma ascites cells

| Glucose (mg.%) | Time of incubation (minutes) | DNP (molar) | Carbon dioxide: Amount ($\mu$ moles) (*a*) | Carbon dioxide: Relative SA (*b*) | Carbon dioxide: Glucose carbon oxidation ($\mu$ atoms) (*a* × *b*)/100 |
|---|---|---|---|---|---|
| 112 | 35 | .. | 10 | 12 | 1·2 |
| (7 mg./6 ml. | | $10^{-4}$ | 17·5 | 45 | 7·9 |
| medium) | | $5\cdot10^{-5}$ | 22·5 | 54 | 12·2 |
| | | $10^{-5}$ | 15 | 38 | 5·7 |
| 100 | 45 | .. | 12·5 | 17 | 2·1 |
| (5 mg./5 ml. | | $8\cdot10^{-5}$ | 24 | 42 | 10·1 |
| medium) | | $4\cdot10^{-5}$ | 20 | 50 | 10·0 |
| | | $8\cdot10^{-6}$ | 16·7 | 32 | 5·3 |
| 167 | 35 | .. | 10 | 27 | 2·7 |
| (10 mg./6 ml. | | $5\cdot10^{-5}$ | 18 | 44 | 7·9 |
| medium) | 60 | .. | 16 | 34 | 5·4 |
| | | $5\cdot10^{-5}$ | 21 | 54 | 11·3 |
| 50(*) | 150 | .. | 57 | 34 | 19·4 |
| (1·5 mg./3 ml. | | $5\cdot10^{-5}$ | 85 | 45 | 38·3 |
| medium) | | | | | |

The reason for the failure to stimulate the oxidation of endogenous substrates in the presence of DNP and excess glucose is not necessarily due to an inhibition of the fatty acid oxidation cycle by DNP but may be due to a competition for coenzymes (diphosphopyridine nucleotide, coenzyme A), as discussed above.

*The effect of glucose and 2:4-dinitrophenol on the oxidation of* [1–$^{14}$*C*] *leucine and* [1–$^{14}$*C*] *glutamate*

It has been reported earlier (Emmelot and van Vals, 1957) that glucose depressed the oxidation of [1–$^{14}$C] leucine and increased that of [1–$^{14}$C] glutamate to carbon dioxide. The latter experiments were conducted under conditions in which a marked pH change of the medium was likely to occur. It has now been found that the oxidation of the two amino acids by the ascites tumour cell is markedly dependent upon the pH. At pH values from 7·4 to 6·0 the oxidation of glutamate was progressively increased whereas that of leucine was decreased. Correcting for the limited pH change which occurred under the conditions of the present experiments (0·02 and 0·03 M phosphate buffer) it was found that glucose (0·006 M) inhibited leucine oxidation for about 20 per cent but had little effect on glutamate oxidation. The fall in protein labelling from [1–$^{14}$C] acetate by glucose observed earlier (Emmelot and van Vals, 1957) might thus have been due to a diminished introduction of $^{14}$C into glutamate as a result of the effect of pH on the equilibrium [1–$^{14}$C]-$\alpha$-ketoglutarate $\rightleftharpoons$ ($^{14}$C) glutamate.

DNP ($10^{-4}$ M) stimulated leucine oxidation 30–75 per cent but depressed glutamate oxidation by 20–40 per cent in the absence of glucose ; these effects were primarily due to changes in the SA but not in the amount of the $CO_2$ produced. These results show that DNP may affect the oxidation of the two amino acids differently.

### SUMMARY

A study of the oxidation of acetate, butyrate, leucine and glutamate, all labelled with $^{14}$C in carbon atom number one, to respiratory carbon dioxide by the $S_3A$ ascites mammary carcinoma in the absence and presence of glucose and/or DNP, and of the oxidation of various concentrations of [U–$^{14}$C] glucose in the presence and absence of DNP, has shown the following results : the fatty acid oxidation cycle is impaired but the oxidation of acetate is not impaired by the presence of glucose ; glycolytic ATP may substitute for mitochondrial ATP in the acetate-activating reaction ; only a fixed amount of glucose is oxidized by the ascites cells under the present experimental conditions, regardless of the concentration of glucose ; the extra $CO_2$ which is produced in the presence of DNP and excess glucose is mainly or exclusively derived from glucose ; leucine and glutamate oxidation are affected differently both by DNP and by changes in the pH of the medium.

### REFERENCES

Bloch-Frankenthal, L. and Weinhouse, S.—(1957) *Cancer Res.*, **17**, 1082.
Chapman, D. P., Brown, Jr., G. W., Chaikoff, I. L., Dauben, W. G. and Fanash, N. O.—(1954) *Ibid.*, **14**, 372.
Crabtree, H. G.—(1929) *Biochem. J.*, **23**, 536.
Dickens, F. and Simer, F.—(1930) *Ibid.*, **24**, 1301.
Emmelot, P. and Bos, C. J.—(1955*a*) *Rec. Trav. chim. Pays-Bas*, **74**, 1343.—(1955*b*) *Experientia*, **11**, 353.—(1957) *Enzymologia*, **18**, 179.—(1959) *Brit. J. Cancer*, **13**, 520.
*Idem*, Bos, C. J., Brombacher, P. J. and Hampe, J. F.—(1959) *Ibid.*, **13**, 348.
*Idem* and Bosch, L.—(1955) *Ibid.*, **9**, 327.
*Idem* and van Vals, G. H.—(1957) *Ibid.*, **11**, 620.
Kun, E., Talalay, P. and Williams-Ashman, H. G.—(1951) *Cancer Res.*, **11**, 855.
Medes, G., Paden, G. and Weinhouse, S.—(1957) *Ibid.*, **17**, 127.
*Idem* and Weinhouse, S.—(1958) *Ibid.*, **18**, 352.
Racker, E.—(1956) *Ann. N.Y. Acad. Sci.*, **63**, 1017.
Slechta, L., Jakubovic, A. and Sorm, F.—(1955) 'Resumées des Communications, 3ième Congèrs International de Biochimie.' (Bruxelles, 1955) p. 126 (14–39).